# Reconstruction of a Real World Social Network using the Potts Model and Loopy Belief Propagation

**DOI:** 10.3389/fpsyg.2015.01698

**Published:** 2015-11-09

**Authors:** Cristian Bisconti, Angelo Corallo, Laura Fortunato, Antonio A. Gentile, Andrea Massafra, Piergiuseppe Pellè

**Affiliations:** ^1^CoSSNA Group, cPDM Lab, Department for Innovation Engineering, University of SalentoLecce, Italy; ^2^EKA srlLecce, Italy; ^3^Advantech srlLecce, Italy

**Keywords:** social network analysis, Potts model, network reconstruction, community detection, loopy belief propagation, inverse problem, quantum structures

## Abstract

The scope of this paper is to test the adoption of a statistical model derived from Condensed Matter Physics, for the reconstruction of the structure of a social network. The inverse Potts model, traditionally applied to recursive observations of quantum states in an ensemble of particles, is here addressed to observations of the members' states in an organization and their (anti)correlations, thus inferring interactions as links among the members. Adopting proper (Bethe) approximations, such an inverse problem is showed to be tractable. Within an operational framework, this network-reconstruction method is tested for a small real-world social network, the Italian parliament. In this study case, it is easy to track statuses of the parliament members, using (co)sponsorships of law proposals as the initial dataset. In previous studies of similar activity-based networks, the graph structure was inferred directly from activity co-occurrences: here we compare our statistical reconstruction with such standard methods, outlining discrepancies and advantages.

## 1. Introduction

A growing interest raised in recent years about policy networks in social and organizational studies: the concept has flourished even in the absence of a widely agreed definition. Among the most successful ones, we may quote (Börzel, 1997) and the concept of horizontal networks linking a variety of actors, who share common interests about a policy, and cooperate toward its adoption. Now, such a broad idea withstood critiques considering the policy network a mere metaphor, more than a model capable of understanding the process of genesis and evolution of policies (Dowding, 1995). A rich literature has adopted both qualitative and quantitative methods to analyse the *network* paradigm. In fact, in most study cases, the relations between the actors involved are depicted as links between the corresponding nodes of a graph (the actors). Most discussions are also driven by network analysis tools and methods (Besussi, 2006).

Among quantitative methods, for our case study we focused on the collaborative nature of policy networks, dealing with *vote behavior*, an idea originally dating back to the “socio-structural and interactional effects,” investigated since (Lazarsfeld et al., 1968). Here, however, following a recent but well developed approach, sponsorships and endorsements of law *proposals* are tracked in the dataset, rather than proper voting behavior when these proposals are approved or rejected. Social Network Analysis (SNA) performed with co-sponsorships and other similar data in legislative bodies was started by Fowler (2006), where the network structure and proximity measures among the US Senate members were obtained. Further analyses were also based on roll calls, but they focused upon the creation and evolution of communities in the network (Porter et al., 2007; Zhang et al., 2008). Retrieving the policy network in a legislative body, and communities therein, using roll-calls and co-sponsorship data, instead of final votes, is a method embedding both advantages and disadvantages. A useful discussion about the point can be found in Chiru and Neamu (2012).

Main interest of this paper is the problem of preliminary reconstruction of member-member networks, starting from the member-activity affiliations, i.e., the roll-call votes data. Indeed, when it comes to the network reconstruction, the preliminary step of most SNA approaches is affected by the simplistic assumption that: two people are related to each other if, and only if, they perform simultaneously a (sub)set of activities, and the strength of their interaction is measured directly counting (and weighting) these co-occurrences.

Evidently, this standard method for network reconstruction can be improved in its capability of finding *hidden* links, or removing those due to noise and bearing no useful information. Various strategies may contribute significantly in this improvement: both those originated from SNA realm itself (e.g., adopting homophily for the study of the network structure); or from other fields (e.g., analysing covariates generated by different observations of the network, making use of random/mixed effects models, or checking covariance data against pseudo-randomization in the samples, etc.). The interested reader may find more details in specific papers. For example, in Newman and Leicht (2007) it is performed the reconstruction of the clusters inside a large-scale network via mixture models, investigating similar structural connections among the nodes. A mixture model in random graphs is used also in Daudin et al. (2008), but this time enriching it with a Bayesian approach, with the purpose to infer unknown classes (Nowicki and Snijders, 2001). Finally, in Jedidi et al. (1997) a general finite mixture structural equation model is built, capable of dealing with heterogeneities in the network's structural equation models, and based upon a set of observed variables (measured with error). In general, these approaches may adopt finite mixture simultaneous equation models, finite mixture confirmatory factor analysis, and finite mixture second-order factor analysis.

Most statistical methods outlined above are a way to relax the strong assumption that filters only those interactions due to co-occurrences. Here, instead, it is discussed an approach adopting the *inverse Potts* model, originated from Condensed Matter Physics. Inverse models aim to infer and model the interactions in an unknown network structure, starting from recursive observations of the nodes' states. As such, these models are adapt to capture underlying quantum structures in a decision making process, whenever the final decision state can be deduced in terms of the observed actions (this argument will be discussed in Section 2). Moreover, this paper also envisages how a Q-states Potts model enables a much better understanding and mimicking of the statistical features of complex network structures, compared for example to a more basic Ising modeling^1^. The approach is tested against a policy network reconstruction, starting from co-sponsorship data collected from the Italian Senate^2^.

It is worth to notice how Ising and Potts (*direct*) models have already found a large number of applications also in the realm of social sciences (Phani et al., 2004; Bordogna and Albano, 2007), including policy networks (Liu et al., 2010), but always applied to networks whose structure had been inferred previously by other strategies. However, the inverse problem formulation has been confined to the Ising model alone, and most of its interest for non-physical problems has involved so far only biological and neural sciences (Yamanishi et al., 2004; Ricci-Tersenghi, 2012), or image reconstruction tasks (Kiwata, 2012). To authors' knowledge, this paper is the first using the inverse Potts problem to *reconstruct* a network in social sciences, and it is in general the first to apply a moment-based Loopy Belief Propagation (LBP) method^3^ to solve the Potts inverse problem in the real world.

The paper will be structured as follows. In Section 2, we will present how the Q-states Potts model intervenes in network reconstruction, and our approach to solve it. Then, in Section 3, a reconstruction of the Italian Senate network is reported, starting from data tracking co-sponsorships of law-proposals and inferring interactions among the senators, according to their decision patterns. Finally, in the Conclusions we will compare the results with traditional SNA methods, i.e., not employing statistical inference.

## 2. Model and methodology

The principle behind the approach described in this paragraph is that (co-participation in) activities of an organization lead(s) to *two-body interactions* among the organization members, and these interactions can be captured by a networked structure. In other words, a complete approach handling relations between different realms (e.g., users and activities) must be able also to examine relations within each realm, separately. Using typical SNA nomenclature, this means computing a *one-mode* network (represented by an *adjacency* matrix), starting from a *two-mode* network (represented by an *affiliation* matrix, that reports participations in the activities, by different organization members). Currently, the standard approach to deduce the one-mode matrix is based upon a mere counting and normalization of co-occurrencies, according to some schemes: these include matches-counting, covariance and correlation measures, cross-products, up to Bonacich and Jaccard indexes (Hanneman and Riddle, 2005). Each of these methods brings along some peculiar features, and the Jaccard index in particular is widely adopted (Borgatti, 2012), being well-suited for sparse affiliation matrices that are very common in the real world.

However, none of these standard approaches resembles probabilistic features, capable of taking into account noisy signals, anti-correlations and co-occurrences of *idle states*^4^. This issue highlights the chance to improve the reconstruction of the corresponding one-mode networks, by a mapping to an inverse statistical problem for pairwise Markov Random Fields (MRF), as discussed in detail later. Especially for large systems, inverse statistical problems are computationally expensive, and approximate methods must be used. For the inverse Ising problem are known: expansions in *correlations* and *clusters* (Sessak and Monasson, 2009; Cocco and Monasson, 2011), methods based upon the *Bethe* approximation (Ricci-Tersenghi, 2012), and *pseudo-likelihood* methods (Ekeberg et al., 2013). Here, we will refer to the moment representation of the LBP approach (MR-LBP), considered particularly advantageous for solving this task (Horiguchi, 1981).

However, Ising models pose severe limitations for SNA applications^5^, and it would be advantageous to switch to a more general Q-state Potts modeling. A theoretical extension of MR-LBP for this general inverse problem has been provided already by Yasuda et al. (2012), making use of an expansion in *Chebyshev polynomials*. This approach is briefly outlined in this paragraph, before explaining how to match it with the specific needs of our case. As the first, however, it is important to discuss at an introductory level why inverse Potts (Ising) modeling are considered adapt to deal with affiliation matrices, that may well derive from quantum features of decision processes.

The starting point of the inverse Q-state Potts problem is a set of *M observations*: D = {*d*^μ^ ∈ {0, 1, …*Q* − 1}^*n*^|μ = 1, 2, …*M*}. The task of the inverse problem is to reconstruct the (Potts) model subtended to the observations^6^. In other words, each observation in D can be considered a “snapshot” of the network at a certain moment in time, where the (positive integer) *state* of each node *x*_*i*_ is observed as $d_{i}^{\mu }$ at the μ-th observation. Pairwise states are indicated as *x*_(*i, j*)_: = {*x*_*i*_, *x*_*j*_}, meaning that, at the time of the same observation, the states of nodes *x*_*i*_ and *x*_*j*_ were found to be as in *x*_(*i, j*)_. In this study case, the allowed *Q*-states can be interpreted as the possible decisions and thus positions (both active or not), about a law proposal, which can be held by the Senate members.

Now, among the fundamental principles of Quantum Mechanics, there is the possibility that if an object can be in either of two generic *orthogonal*^7^ states |ϕ〉 and |ψ〉, then, in general it is also allowed to be in any linear superposition of the two: α|ϕ〉 + β|ψ〉. Intuitively, however, when a measurement of the object's state is performed, the state must collapse into either one *or* the other. This is also at the core of many models exploiting quantumness in the cognitive realm (Haven and Khrennikov, 2013). Mapping this general statement into our specific study case is equivalent to supposing that policy network agents perform decisions according to the same scheme of a quantum state measurement. Intuitively, this means that these agents do not already “embed” a decision about what to do, before being asked support for a roll-call. Only when they are confronted with the decision making, they *contextually* choose one of the possible alternatives to act: before that moment, it is possible to suppose they were in a superposition of some (all) possible decisions. I.e., they were considering also alternatives, before finalizing their choice.

More formally, the generic decision state of each senator can be mapped as a superposition state |*X*_*i*_〉, in (some of) the Q-states |χ〉 of the Potts model:

(1) $$\begin{array}{l} |X_{i}\rangle =\sum_{\chi =1}^{Q}\beta_{i,\chi }|\chi \rangle \end{array}$$

and each observation of a node's state can be understood as a POVM of |*X*_*i*_〉 in the basis of the states |χ〉, that are mutually orthogonal. This underlies the plausible assumption that a single member may *desire*—but not *intend*—more than one decision at once, toward a certain law proposal: for example they cannot simultaneously support and ignore the same roll call. Non-classical effects of this superposition of states guiding the final decision have already been discussed, e.g., in Aerts et al. (2012), and a more complex quantum modeling of decision making has been proposed in Bisconti et al. (2015).

It may be noticed that, when introducing at first the Potts model in this paragraph, no explicit reference to quantum states was made. In fact, this is because an effective treatment of the quantum Potts model can be done within a classical formalism: a more technical justification follows in the rest of this paragraph. Indeed, a quantum Potts model introduces a Hamiltonian characterized by two-body^8^ interactions as:

(2) $$\begin{array}{l} H_{Potts}=-\underset{\left\{i,j\right\}}{\sum }H_{\left(i,j\right)}\underset{\chi }{\sum }P_{i}^{\chi }P_{j}^{\chi } \end{array}$$

where $P_{i}^{\chi }$ are projectors onto the |χ〉 state of the local space for the *i*-th node. *H* is instead called the *ferromagnetic coupling*, and it captures the intensity of interaction among the nodes.

It is known how any classical (finite-dimensional) spin model on a lattice can be associated to a quantum model (Somma and Ortiz, 2010), defined on the same lattice, by mapping every classical state *x*_*i*_ into measurement outcomes of the state |*X*_*i*_〉 and viceversa. Classically, the spin model has an energy functional that is:

(3) $$\begin{array}{l} {ℰ}_{Potts}=-\sum_{\left\{i,j\right\}}H_{\left(i,j\right)}x_{\left(i,j\right)} \end{array}$$

Therefore, the energy functional maps into the eigenvalues of the Hamiltonian operator defined in Equation (2), and when performing statistical inference from the observations of the nodes' states in the network, this correspondence allows us to refer directly to the values of the classical variable *x*_*i*_. In the following, therefore, the baseline assumption will be that a model subtending a statistical treatment of the network reconstruction problem, inspired by a quantum-mechanical counterpart, can be far more efficient in revealing hidden links and patterns from observations, inferring even those interactions that standard methods are not capable of detecting.

### 2.1. The inverse potts model

It has been seen how a statistical approach to the Potts problem, dealing with classical variables *x*_*i*_, still implicitly underlines an intrinsically quantum process of decision making, because the likelihood of observing a certain value *d*_*i*_ for *x*_*i*_ can be interpreted in terms of projecting the generic quantum state |*X*_*i*_〉, onto the corresponding basis state |χ〉, where each of the orthogonal basis states identifies a single possible decision. This paragraph is devoted to a detailed explanation of the algorithm inferring relationships among nodes, from the set of observations performed: non-technical readers may skip it and move to the considerations in Section 2.2.

It can be observed how the probability distribution—for observations of the node states *x*—is clearly connected with the energy functional in Equation (3):

(4) $$\begin{array}{l} P\left(x\right)\propto \exp\left[-\overset{n}{\underset{\left(i,j\right)\in E}{\sum }}H_{\left(i,j\right)}\left(x_{\left(i,j\right)}\right)\right] \end{array}$$

and this closely resembles the probability distribution in general pairwise MRF formalism. *E* defines here the set of connections expected in the model, and therefore the condition (*i, j*) ∈ *E* set in the summation can be understood as an explicit network constraint, whereas in Equation (3) we had the generic {*i, j*}. Now, in the inverse problem, the *H*_(*i, j*)_ setting up the network model are unknown^9^ and must be inferred by the probabilities in Equation (4). In terms of the orthogonal set of Chebyshev polynomials Φ_*k*_(*x*_*i*_) and appropriate constants $J_{\left(i,j\right)}^{\left(k,l\right)}$, it is possible to write the two-body potential function *H* as:

(5) $$\begin{array}{lll} H_{\left(i,j\right)}\left(x_{\left(i,j\right)}\right)= & \frac{1}{\sqrt{Q}}\overset{Q-1}{\underset{k=1}{\sum }}\left[J_{\left(i,j\right)}^{\left(k,0\right)}\Phi_{k}\left(x_{i}\right)+J_{\left(i,j\right)}^{\left(0,k\right)}\Phi_{k}\left(x_{j}\right)\right]+\; & \;\\ +\overset{Q-1}{\underset{k=0}{\sum }}\overset{Q-1}{\underset{l=0}{\sum }}J_{\left(i,j\right)}^{\left(k,l\right)}\Phi_{k}\left(x_{i}\right)\Phi_{l}\left(x_{j}\right)+constant\; & \; \end{array}$$

where constant terms in the expansion (e.g., Φ_0_(*x*_*i*_)) have been all included in the last constant term. Starting from Equation (5), Yasuda et al. (2012) applied a moment representation of the LBP scheme and *message-passing* rules to the MRF described so far. Within the *Bethe* approximation, it was shown how it is possible to approximately find the constants *J* from marginal probabilities of the observations:

(6) $$\begin{array}{l} J_{\left(i,j\right)}^{\left(k,l\right)}=-\overset{Q-1}{\underset{x_{i}=0}{\sum }}\overset{Q-1}{\underset{x_{j}=0}{\sum }}\Phi_{k}\left(x_{i}\right)\Phi_{l}\left(x_{j}\right)\ln\;P_{\left(i,j\right)}\left(x_{\left(i,j\right)}|D\right) \end{array}$$

thus minimizing the (Bethe) approximate entropy of the model: the P probability values are used to reconstruct the parameters of the Potts model.

The probabilities P, for observing in D, respectively values *x*_*i*_ and *x*_(*i, j*)_, can also be expressed as sums of Chebyshev polynomials:

(7) $$\begin{array}{l} P_{i}\left(x_{i}|D\right)=\frac{1}{Q}+\overset{Q-1}{\underset{k=1}{\sum }}{\langle \Phi_{k}\left(x_{i}\right)\rangle }_{D}\Phi_{k}\left(x_{i}\right) \end{array}$$

(8) $$\begin{array}{lll} P_{\left(i,j\right)} & \left(x_{\left(i,j\right)}|D\right)=\; & \;\\ \frac{1}{Q^{2}}+\frac{1}{Q}\overset{Q-1}{\underset{k=1}{\sum }}\left[{\langle \Phi_{k}\left(x_{i}\right)\rangle }_{D}\Phi_{k}\left(x_{i}\right)+{\langle \Phi_{k}\left(x_{j}\right)\rangle }_{D}\Phi_{k}\left(x_{j}\right)\right]\; & \;\\ +\overset{Q-1}{\underset{k=1}{\sum }}\overset{Q-1}{\underset{l=1}{\sum }}{\langle \Phi_{k}\left(x_{i}\right)\Phi_{l}\left(x_{j}\right)\rangle }_{D}\Phi_{k}\left(x_{i}\right)\Phi_{l}\left(x_{j}\right)\; & \; \end{array}$$

Here, the interesting advantage of using the LBP moment representation is that all the quantities 〈…〉_D_ can be derived by averaging over an appropriate number of *M* observations D of the network.

It can be both intuitively predicted, and numerical experiments in Yasuda et al. (2012) confirmed it, that the number of observations used is correlated with the quality of the final network reconstruction obtained. It shall be observed how in the original paper, numerical experiments were limited to the case when the network structure underlying the inverse problem was a non-periodic lattice (i.e., |*i* − *j*| ∉ {θ[min(*i, j*) mod *p*], *p*} ⇒ (*i, j*) ∉ *E* ⇔ *J*_(*i, j*)_ = 0, where θ the step function and *p* the lattice period).

Considering that the main specific interest of this paper is the reconstruction of the network, i.e., the pairwise interactions among the nodes, here the key parameter in the Potts model is indeed *H*_(*i, j*)_, measuring the intensity of connection between users *i* and *j* in the network. Equation (5) shows that *H*_(*i, j*)_ is directly related to the set of constants $J_{\left(i,j\right)}^{\left(k,l\right)}$.

An interesting feature, that contributes to the sensitivity of this approach compared to standard ones listed above, is that Φ_*k*_(*x*_*i*_)Φ_*l*_(*x*_*j*_)—used in Equation (6) for calculating *J*—is in general different from 0, even when *k* ≠ *l*. Therefore, interactions are inferred also when simultaneous participation in the same activity plays no role. The interpretation is that, even if one expects no interaction to occur among users because they tended to perform different activities^10^ in the observation snapshots, this assumption is actually tested by the reconstruction method against the observations, and indirect (“out-of-diagonal”) correlations may be detected.

As better explained in Section 2.2, in most cases data collected from social networks require caution before being used as “observational data” in a Q-state inverse Potts problem. Therefore, it is interesting to mention the possibility to simulate observations, whenever data about the probability distributions are known to depend upon some parameter(s). For example, observation samples may be reconstructed using one only parameter α in a *generative* model: in this case probabilities of observing a certain collective state *x* are computed according to α, and it is possible to write down averaged functions (such as the averages required by Equations 7 and 8):

(9) $$\begin{array}{l} {\langle f\left(x\right)\rangle }_{D}=\underset{x\in D}{\sum }f\left(x\right)P_{GenMod}\left(x|\alpha \right) \end{array}$$

Clearly, because of the assumptions underlying the LBP inverse problem approach, the most general choice for the generative model (*GenMod*) must be a Q-state Potts model.

### 2.2. Data and observations

It is left to explain how to employ an inverse Q-state Potts model for the reconstruction of the Italian Senate network of members, starting from data tracking law co-sponsorships by senators. In the Italian legislative system, a law undergoes a few preliminary steps before being discussed in the Senate. As the first, one or more^11^ senators are responsible for writing it down, signing and proposing it; these are very similar to the *sponsors* in US legislative system. After that, other senators who are aware that this specific law is being proposed for discussion, may co-sign it, as an act of endorsement. They act as the US *co-sponsors*. According to the Senate's schedule, the law is then discussed in detail and subjected (eventually) to a final vote. Therefore, collecting (co)sponsorships' data brings along a considerable insight about patterns of collaboration and support among the senators, and can be considered equivalent to other studies performed with similar legislative bodies in other countries, as cited in the introduction.

Our case study focuses on the first *part*^12^ of the XVI Italian legislature, using co-sponsorship data for Senate roll calls in the same period. We chose this period for two reasons. As the first, one of the intents is to find communities (and their members) in the network by automatic community detection algorithms, and compare the resulting groups with the “official memberships in political parties” of the senators. For this purpose, the beginning of a legislature is ideal, because senators have just been elected as members of a certain political party^13^. This makes it easy to refer to these parties as their *true memberships*, whereas at later points in time, several senators may have moved to different political parties (e.g., because some parties have been dismantled), and tracking these changes in a mindful way turns extremely difficult. Moreover, the dataset of this study case is the most recent (thus eventually more interesting from a policy network point of view), while referring at the same time to a past Legislature. This renders available data “crystallized,” with less risk of updates to occur.

Usage of minimization procedures in Potts-like models for legislative bodies is not fully new in the literature: for example, in Liu et al. (2010), an Ising model had been used to model the US Senate network starting from bill cosponsorships. However, compared with this previous study, there are here a few important differences.

- In Liu et al. (2010), the quantum Ising model is *not* used for the network reconstruction, achieved by a simple weighted interaction counts procedure. The Ising model intervenes merely in a second phase, for the influence maximization analysis.
- Because of the intrinsic political nature of the Italian VS the US Senate, whereas a 2-state Ising model is perfectly adapted to the strongly bipartite US case, it is rather limiting when used to describe the multi-partite structure of its Italian counterpart, that requires a more generic Q-states approach.
- Observing more closely the available data, US co-sponsorship data of the 108th Congress (used for the network analysis in Fowler, 2006 and derived ones) had in average 285 bills (co)sponsored per legislator—against 62 bills/legislator for Italian co-sponsorship data from the XVI legislation. Each US bill was (co)sponsored in average by 4 legislators—while 8 legislators per bill was the average in the Italian case. The total is of 4630 bills for 100 senators in the US case, and 3100 (*M*) law proposals for 338 (*N*) senators in the Italian case. Summarizing, the US Senate was much more active in sponsoring bills, and still proportionally more active in co-sponsoring, when compared to the Italian counterpart.

The connectedness of the US legislative network, given by the ratio cosponsorships/senators, and the reduced number of communities therein, make it adapt of being treated with an Ising 2-state model. Also standard methods may reproduce the structure of that network in an acceptable way, given that its high density may well represent^14^ the absence of hidden or evolving links. This considerations, however, suggest that the same approach may provide poor results for the Italian situation.

Here we intend to use a Q-state Potts model *directly* for the network reconstruction, as outlined in Section **??**. A naive application of the model may involve two only possible states for the nodes (senators).

1. An *active* state (*x*_*i*_ = 1), corresponding to nodes sponsoring or co-sponsoring a bill, when this is being proposed or introduced. It is indeed intuitive to consider the request for cosponsorships an observational event, measuring the behavior of the nodes, and therefore the state they are in.
2. A *passive* state (*x*_*i*_ = 0), when the nodes do not act as (co)sponsors when a bill is introduced (i.e., they are detected as inactive when they undergo “measurement”), and therefore no endorsement is tracked in the data.

This would be equivalent to an Ising model. In order to better explain what follows, it is worth a parenthesis about the Italian case. It was highlighted how each *activity*, i.e., the proposal for each law in the Senate, was participated in average by about 8 people (*N*_*A*_). That is, for each of the *M* bills, the active community was in average 2.5% of the whole Senate. Now, using a 2-state Potts model as above implicitly generates correlations also among senators often detected in passive states: Indeed, it is evident how co-occurrences of inactive states would be assigned the same importance, in principle, as co-occurrences of active states.

Is this meaningful? Consider the underlying phenomenon: co-endorsing a law proposal presumes a much more intensive link between two senators (as it obviously brings along the sharing of the same political point of view, as well as some sort of acquaintance with the senator who conceived the law itself), compared to simultaneous abstaining from the endorsement (which may be due to lack of chance to discuss and share the law proposal; or to early abundance of cosponsors, making worthless for other senators to join the cosponsoring group; etc.). A simple abstinence from action is an ambiguous behavior, as it supposes no direct opposition or lack of interest. Therefore, it is intuitively necessary to find a mechanism that keeps these *inactive correlations*^15^ less significant, compared to those due to simultaneous observation of the same active state in two nodes^16^.

A first approach may be to still use *Q* = 2, while explicitly ignoring inactive correlations when computing the interaction parameters. This can be done by replacing:

(10) $$\begin{array}{l} x_{i\left(j\right)}\in \left\{0,\ldots ,Q-1\right\}\to x_{i\left(j\right)}\in \left\{1,\ldots ,Q-1\right\} \end{array}$$

in the sums of Equation (6), where we supposed that *x*_*i*_ = 0 corresponds to the only inactive state. However, this choice will miss the chance of capturing hidden connections, due to simultaneous occurrence of inactive states for some specific reason, and particularly the hostility against the law proposal under discussion.

An effective solution, but computationally expensive, is to pick a high enough *Q*-value for the model, assigning different *x*_*i*_ ≠ 1 to members in inactive states. In particular, to avoid aprioristic considerations about the level of interaction of people belonging to the same faction, the random probability of assigning two nodes to the same *inactive* state (*p*_*ina*_) shall not be bigger than the average empirical probability of two nodes being assigned to the same *active* state (*p*_*act*_). Now: $p_{ina}=\frac{N-N_{A}}{\left(Q+1\right)N}\leq p_{act}≅0.024$, which gives in turn: *Q* ≥ 40. Because of the computational complexity of the procedure (*O*(*Q*^2^)), here for demonstrative purposes it will be shown how the performance of the method can change moving from *Q* = 2 up to *Q* = 10. That is, we start by assigning to inactive correlations the same importance as active correlations, then we progressively reduce the importance of the second compared to the first ones. The case with *Q* = 5 has a specific underlying reason: community detection algorithms revealed 5 clusters in the Senate network, when run against the network, reconstructed with the standard Jaccard approach, see Section 3. The intent is therefore to try using this information as an initial guess for the LBP approach, introducing a number of possible states corresponding to community membership (under the reasonable assumption that such a membership strongly influences the co-sponsorship decisions). However, it should be emphasized here that partitioning the network in 5 communities may be non-optimal. Indeed, along the period of the analysis performed, it is true that the Parliament involved 4 major parties, plus senators being independent, or belonging to small^17^ parties, but the 4 major parties were actually joint in 2 different alliances, thus reducing the number of effective communities to only 3. This is an important consideration, therefore it will be discussed again in the following.

There is still another feature in the procedure, left to discuss: cleaning and eventually generating the observation samples. This feature can be tuned as well, in order to introduce aprioristic knowledge about the network structure. In general, there are at least three different strategies to use properly the collected data:

1. a full *generative* model, where at first some standard method is applied to reconstruct the network, this network is used as an initial guess for the interactions among the nodes, allowing to sample a set of observations;
2. a *semi-observational* model, where the observations collected are used directly as samples, but sample averages are adjusted against the network reconstructed via standard methods;
3. a pure *observational* model, that is agnostic of any coarse-grain network structure, and applies directly the LBP procedure to the data: here sample averages are computed directly from the data (that can thus be confused with the observation samples).

It is worth to notice how the first strategy replaces real data with samples obtained according to some reasonable^18^ assumptions about the strength of relationship among nodes, summarized as α_*ij*_ elements^19^ of a preliminary adjacency matrix. For example, in Liu et al. (2010) a count of co-occurrences of active states was used:

(11) $$\begin{array}{l} \alpha_{ij}=\underset{\mu }{\sum }\frac{\delta \left(x_{i}^{\mu },x_{j}^{\mu },1\right)}{n_{\mu }} \end{array}$$

weighted with the number of cosponsors *n*_μ_, for each bill μ.

Even if a standard method is used for the preliminary calculation of the interaction among the nodes, the LBP procedure still intervenes in allowing to infer hidden connections, not evident from the first step. The generative approach is particularly useful whenever only a few or only aggregate^20^ data are available for the analysis. However, this strategy still introduces a manipulation of original data, in order to make the network reconstruction possible or less noisy^21^.

The semi-observational strategy can be seen as a compromise between the adoption of a generative model, and the direct usage of data with no further adjustments. In this case, observation data are used directly for each step of the network reconstruction, except the calculation of averages. More technically, in this case 〈Φ_*k*_(*x*_*i*_)〉_D_ and 〈Φ_*k*_(*x*_*i*_)Φ_*l*_(*x*_*j*_)〉_D_ are not simple averages from the samples' set, but they are adjusted according to Equation (9). As an example, the *P*_*GenMod*_ probabilities of occurrence of the state *x* may be chosen as:

(12) $$\begin{array}{l} P_{Potts}\left(x|\alpha \right)\propto \exp\left[\frac{1}{2}\overset{N}{\underset{i=1}{\sum }}\overset{N}{\underset{j=1}{\sum }}\alpha_{ij}\delta \left(x_{i},x_{j}\right)\right] \end{array}$$

Generally speaking, the introduction of a subtending model as in Equation (12) favors a reconstruction similar to the output of the standard preliminary reconstruction method, because the probabilities of observing configurations (not) matching the standard reconstructions are increased (decreased), compared to the probabilities calculated directly from the observations.

The α parameters were evaluated here in terms of frequencies of matching activities, within the set of observations, according to different approaches. One possibility is a pure frequentist probability, for the two nodes *i* and *j* to be observed in the same *active* state:

(13) $$\begin{array}{l} \alpha_{ij}=\frac{1}{M}\underset{\mu }{\sum }\frac{\delta \left(d_{i}^{\mu },d_{j}^{\mu },1\right)}{n_{\mu }} \end{array}$$

with generalized Kronecker δ(*i, j, k*) = 1 ⇔ *i* = *j* = *k* and null otherwise, and the same weighting of Equation (11). This strategy penalizes the interactions of those nodes having a poor participation rate.

A second derivation for α, instead, was adjusted against the number of times the two users were active:

(14) $$\begin{array}{l} \alpha_{ij}=\frac{1}{\sum_{\mu }\left(\delta \left(d_{i}^{\mu },1\right)+\delta \left(d_{j}^{\mu },1\right)\right)}\underset{\mu }{\sum }\frac{\delta \left(d_{i}^{\mu },d_{j}^{\mu },1\right)}{n_{\mu }} \end{array}$$

thus reducing the bias of the previous formula toward active nodes.

Finally, when a pure observational method is used, the α parameter should play no role^22^, because no generative model needs to be provided and all the averaged quantities are computed as from the original set of data. Unfortunately, a pure observational method with the considered dataset (characterized by *Q* = 2, because of lacking information) intuitively requires to omit the contribution of inactive correlations, such as in Equation (10), in order not to overestimate their contribution.

Whatever the strategy chosen to derive observation samples from original data, the interactions *H*_(*i, j*)_ will be calculated replacing in Equation (5) the pairwise interactions *J* from Equation (6).

## 3. Results and discussion

It was envisaged the importance of the LBP inference method, for discovering non-evident links and connections among the network members, as compared to traditional methods not employing statistical inference. This paragraph illustrates the first numerical application of a LBP procedure, to reconstruct a generic graph Potts model. Previous simulations (Yasuda et al., 2012), indeed, dealt only with lattice-like Potts models: the sums in Equation (12) had a constant α instead of α_*i, j*_, and the allowed indexes were only those compatible with the lattice structure (*i, j*) ∈ *E*.

**Figure 1:**
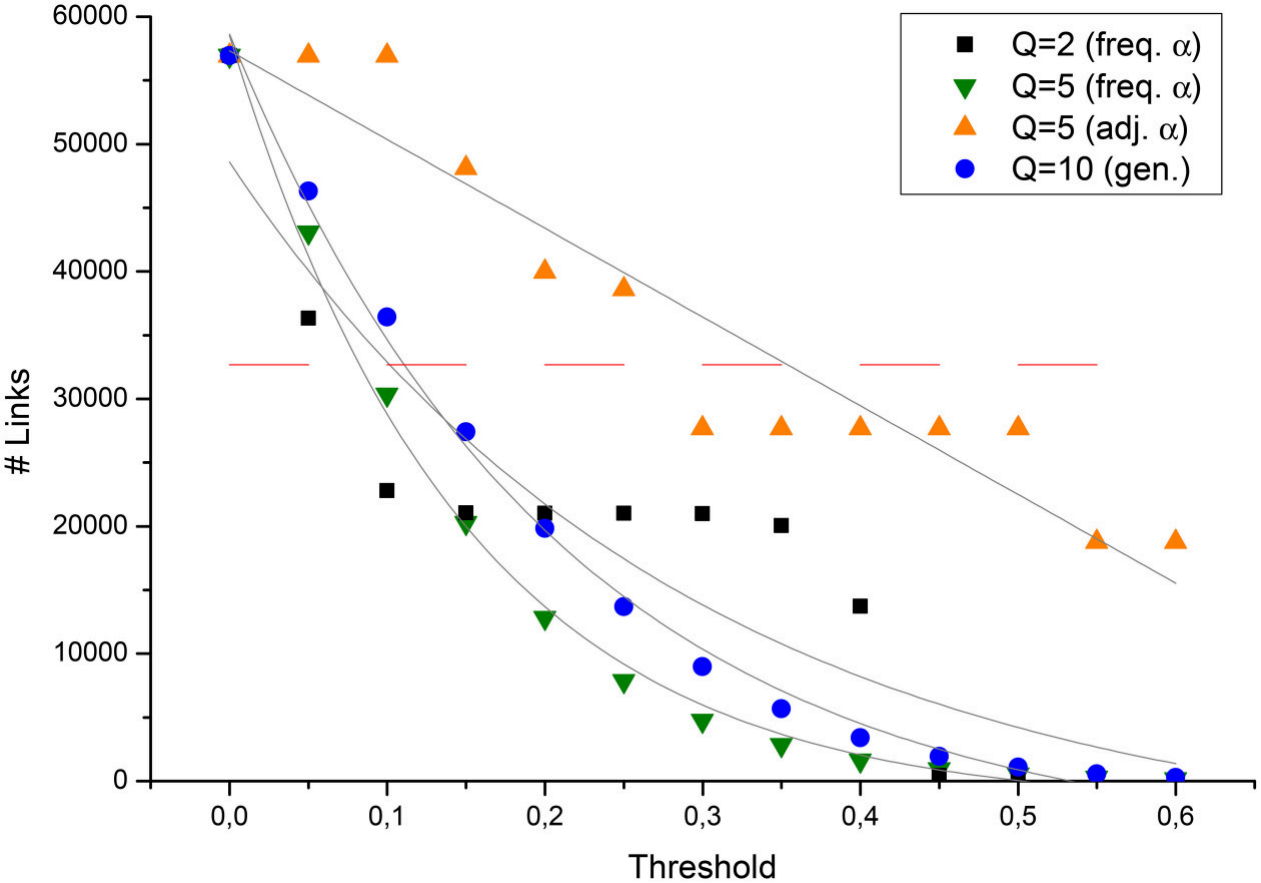
**Number of links detected by the Potts-LBP approach in the original graph, against the ***t***_***m***_ threshold parameter, for some of the reconstruction approaches discussed in the text**. The dashed red line indicated the number of links detected with the Jaccard method.

The first and most important results to be observed are in Table 1 and in Figure 1. In the table are reported the main network parameters for the various methods listed in Section 2. As a comparison, the network was also reconstructed via the *Jaccard index*, a standard method particularly adapt to sparse networks (Borgatti, 2009), such as the one analyzed in this paper (the calculated density is indeed smaller than 0.01). For LBP-reconstructed networks, we introduced an additional parameter, the *threshold* (*t*_*m*_). In fact, after normalizing the intensity of connections (i.e., 0 ≤ *H*_(*i, j*)_ ≤ 1), the density of these networks was close to 1 in most approaches. This is an effect of the sensitivity of the LBP method, prone to reproduce in the final *adjacency* matrix also links due to noise. In order to exclude the weakest links, we set a threshold value *t*_*m*_ = 0.5, thus comparing the residual links with the standard network. It is evident how in all cases, also the LBP-reconstructed social network displays many more connections compared to the Jaccard one. These *hidden* connections would be hard to identify without referring to an inference statistical method, and this is a novelty of the approach. In the pure observational case, because *off-diagonal* interactions were neglected (i.e., the case in Equation 10), also noisy connections tend to occur in a small range, thus producing still a very high density at *t*_*m*_ = 0.5. Because of the increased difficulty to filter properly this noise, the pure observational model will be omitted from analyses in the following. Also the average strength of *all* the links detected “Avg. *H*_(*i, j*)_” has an interesting behavior: it is strongly affected by the initial guess for the network structure, that the LBP method tries to reproduce, and considerably less, instead, by the value chosen for *Q*.

**Table 1:** **Collection of fundamental model and network parameters, for a set of network reconstruction methods, and basic metrics resulting from the analysis**.

**Method**	**# Samples**	***Q***	**Avg.** α	**Density of reconstructed network (*t*_*m*_ = 0.5)**	**Avg. *H*_(*i, j*)_ (normalized)**
Jaccard index	3100	NA	NA	0.00202	0.04901
Generative	100,000	5	0.65984^−04^ (Equation 13)	0.01554	0.16336
	100,000	10	″	0.01907	0.16938
Semi-observational	3100	2	0.65984^−04^ (Equation 13)	0.00540	0.17612
	3100	5	″	0.00936	0.13385
	3100	5	0.01650 (Equation 14)	0.48623	0.50464
Pure observational	3100	2	0.01^*^	0.99275	0.98301

*The analysis with Q = 10 required to artificially generate more samples than the direct observations from data, for the results to be reliable, therefore it is listed only within the Generative case*.

* *For numerical convergence reasons, in the Pure observational case, it was set α_ij_ = constant = 0.01*.

In Figure 1, instead, it is performed a more systematic analysis of the relation between the number of links in the network, against the threshold parameter^23^. A few interesting features are evident. As the first, all statistical methods tend to saturate the network at low values of *t*_*m*_. Moreover, a smoothing effect in the dependency of the number of links on the threshold value is observed, both when increasing *Q* or decreasing the average α_*ij*_. The higher the smoothing, the closest are the data to the expected exponential decay in the number of detected links^24^. Several possible explanations for this conclusion may be proposed. As the first, preliminary community detection analyses with networks reconstructed via standard methods identified 5 groups^25^ in the Italian Senate network. This suggests how the observation of only 2 states with the roll calls tends to produce distortions and artifacts. In fact, results are improved also by randomly introducing states other than the observed (non)sponsoring. Smaller values of α, instead, allow the method to compute the $J_{\left(i,j\right)}^{\left(k,l\right)}$ *not* in proximity of critical values $ln\left(1+\sqrt{Q}\right)$ of the Potts model, thus improving the stability of the results.

**Figure 2:**
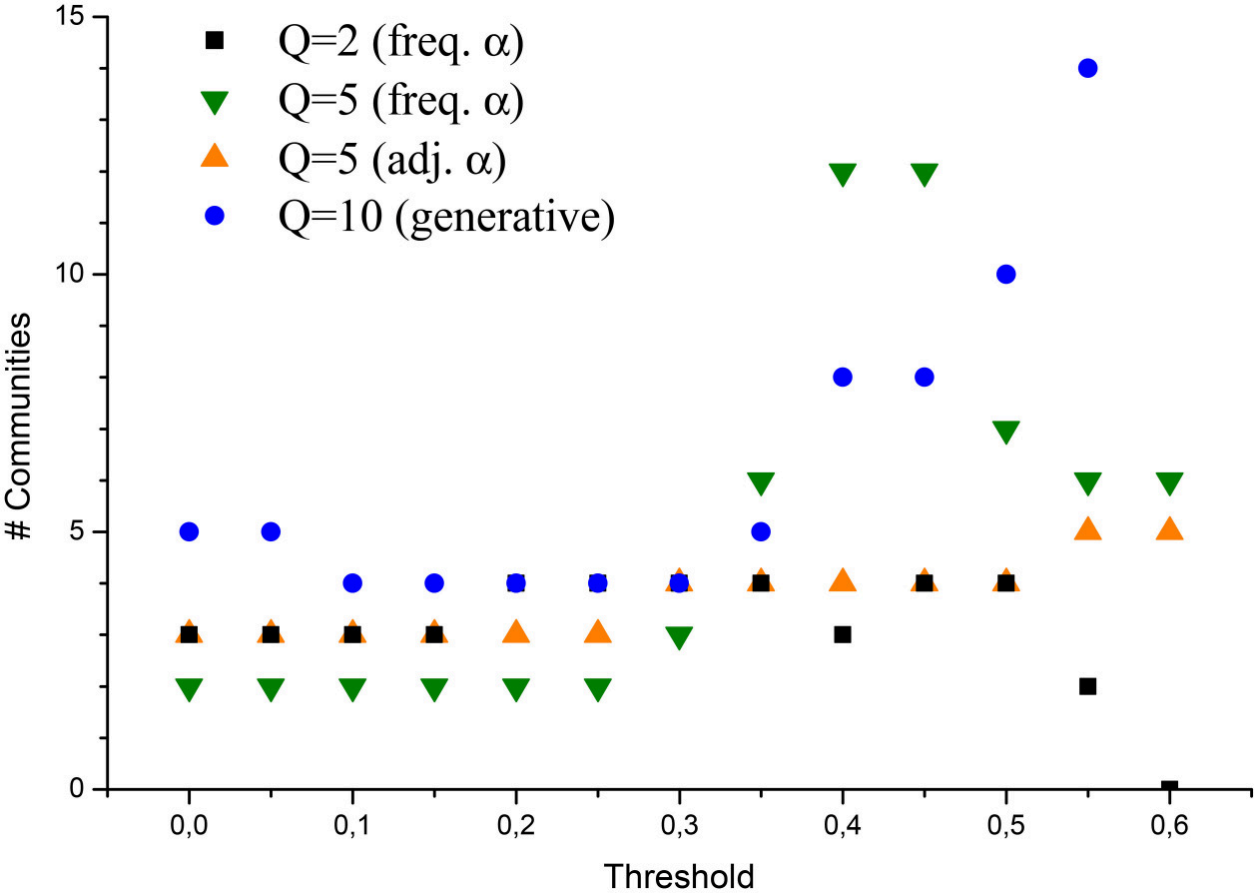
**Number of communities detected in the network via the CNM algorithm, applied to various LBP reconstructed networks**.

A different analysis was focused about the capability of the method not only to reconstruct pairwise interactions, but also to better identify the clusters inside the network, to be interpreted as communities of members. In Figure 2 it is investigated how the number of such communities depends on *t*_*m*_: the plot shows that when a small *t*_*m*_ is taken into account^26^, LBP reconstructed networks have a cluster structure involving 2–3 groups. A plausible interpretation is that, if hidden links are considered, slight differences in the policy approach by the Senate members are swiped out in the analysis, and the CNM algorithm tends to detect only the fundamental communities: the ones related with the party(ies) participating in the Cabinet, and the group of parties opposing the first ones (plus eventually a third group which may be considered as composed by *neutral* senators). As the threshold is increased, and the graph becomes more disconnected, also clustering features are emphasized, and the number of detected communities increases. In particular, when a high number of possible states is allowed (high *Q*), and at the same time weak interactions are hypothesized (α_*ij*_ is small in average), the number of communities tends to “explode.” However, excluding this extreme case, detected communities are otherwise stable, ranging between 3 and 6. It is also evident how, when links in the network are filtered and the cluster structure emerges, the network assuming *Q* = 2 totally fails to reproduce a plausible number of communities: this is clearly due to the artifact of imposing a naturally bipartite network in the model, which does not correspond, though, to the expected network structure.

Finally, we investigated if—and how much—the LBP algorithm is able to improve the assignment of senator-nodes to the “right” political community: i.e., the one identified by the same political party, the senator officially belonged to. The figure of merit will be a sort of a *false discovery rate* (FDR), that is, the ratio between the number of senators assigned by the CNM algorithm to wrong communities, and the total number of senators analyzed. In order to emphasize the role of hidden links, the focus will be the capability of the algorithm to classify the senators as belonging to the group supporting the government, the opposition group, or the mixed independent group.

**Figure 3:**
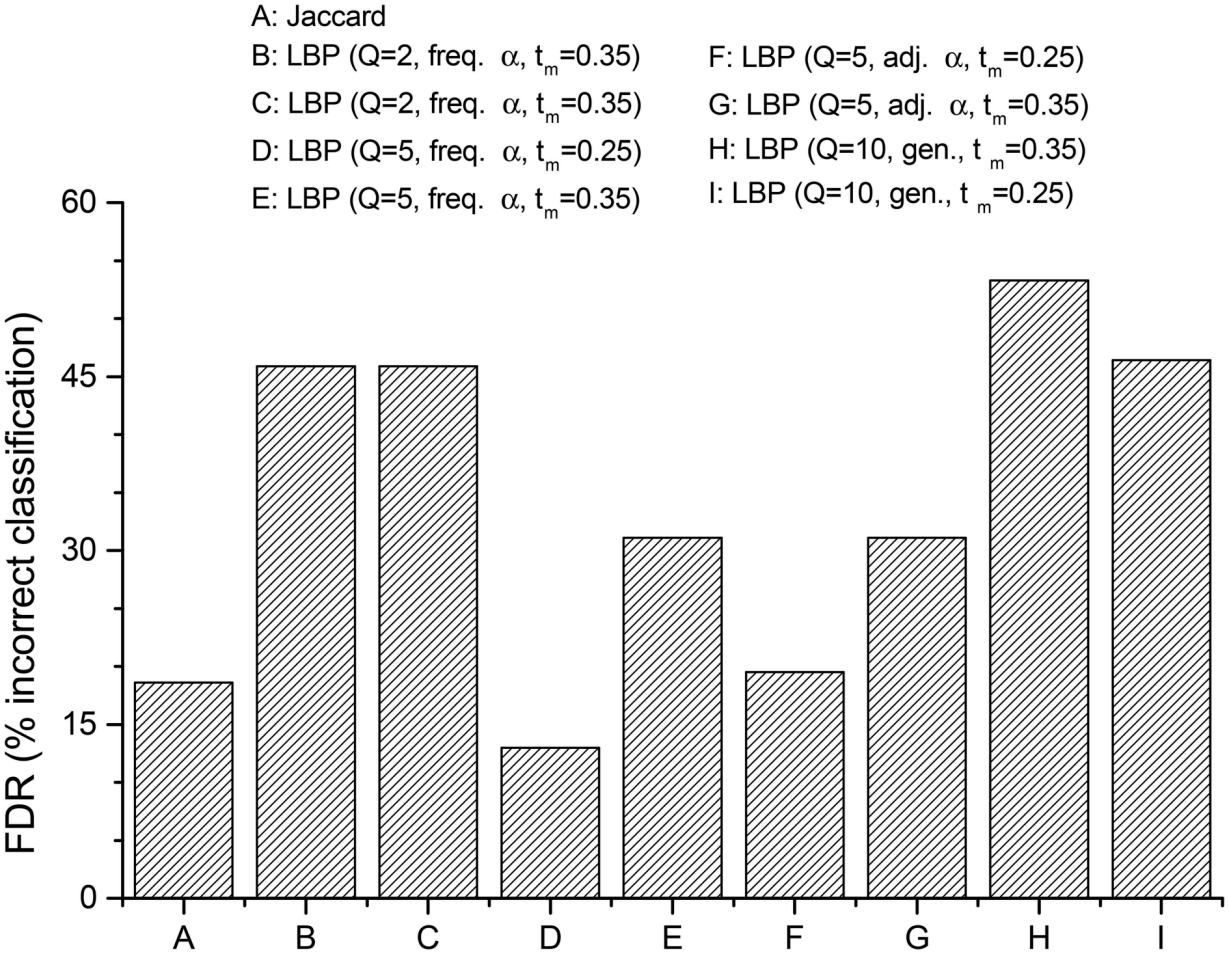
**Percentages of senators mistakenly classified in the “wrong” Senate political community (FDR, see Section 3), for different network reconstruction methods**. Jaccard-reconstructed network is reported for reference. Parameters used for each case are in the Legend.

Referring to Figure 3, the reconstruction of the network via a simple Jaccard coefficient in this case is already capable of reproducing accurately the true membership of the nodes, scoring only about 15% of nodes classified. The case of LBP with *Q* = 2, instead, is very inefficient: almost half of the nodes is misclassifiedm, even if the target number of communities is close enough to allowed values of *Q*. This result shows the importance of extending the Ising model used elsewhere in analyses of policy networks: even when a subtended bipartite interaction is tracked (i.e., sponsoring VS abstaining in a roll call vote), in the end this is a projection of a more complex state, each agent in the network is before performing the voting action. From a modeling perspective, such a gap between model and reality can be reduced by a full quantum Ising model (as showed by Liu et al., 2010), or by semi-classical approaches with a Q-state Potts model. In fact, results with *Q* = 5 display a great improvement compared to the case with *Q* = 2. Especially when *t*_*m*_ has a value in the range where the number of detected communities is stable, the percentage of nodes classified in the wrong group almost matches, or even outperforms the Jaccard one (13%), without assuming a-priori that indirect correlations among the network members are negligible. Interestingly, the best results are achieved for values of the threshold, corresponding to intervals where the number of comunities detected is stable (compare with Figure 2).

**Figure 4:**
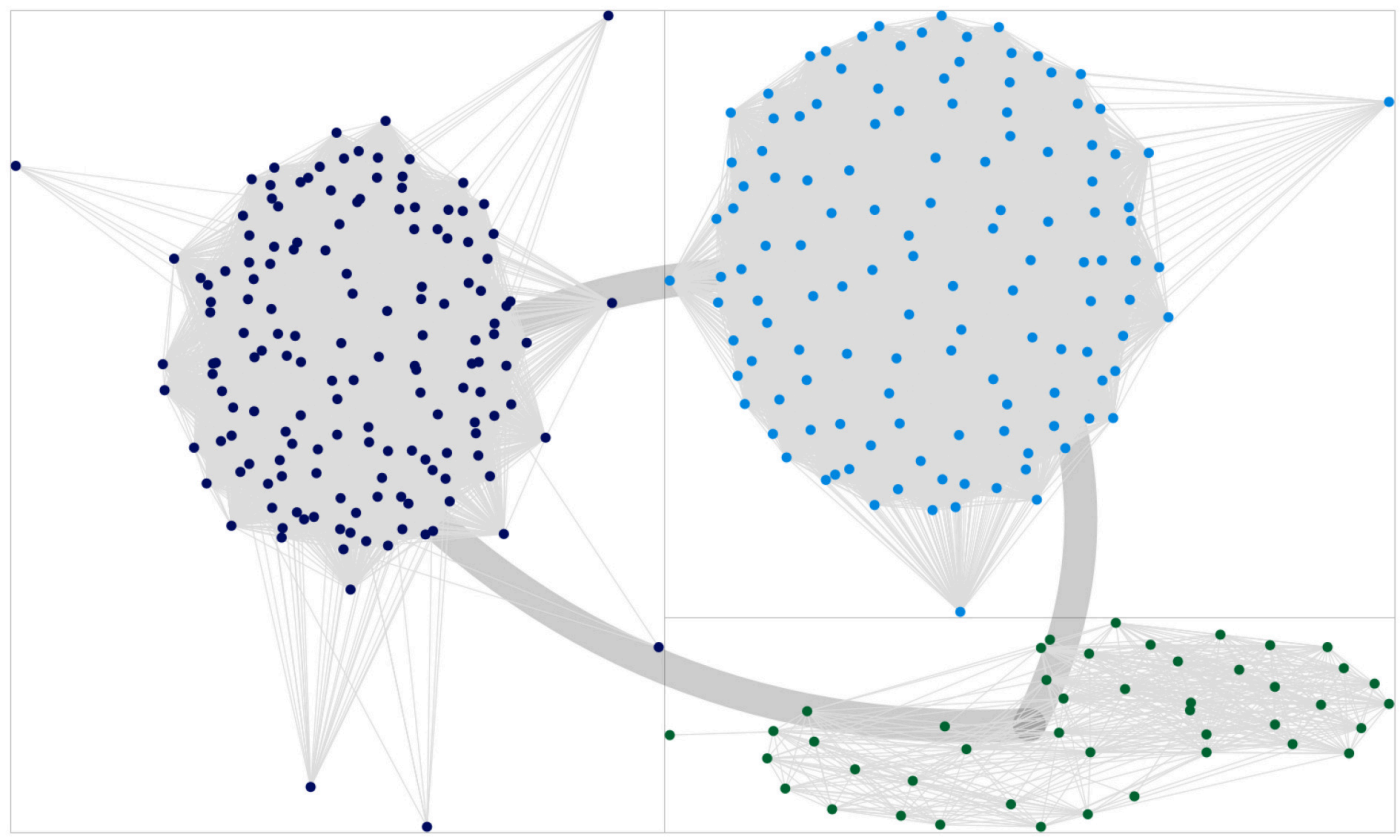
**Plot of the clusters obtained via the CNM algorithm, for the network reconstructed using the standard Jaccard method**. Thick lines connecting the quadrants indicate the global cumulative strength of inter-community links. Dark blue dots indicate the community interpreted as “loyal to the Cabinet,” light blue dots are connected with the “opposition,” green dots are to be interpreted as “indipendent senators.”

**Figure 5:**
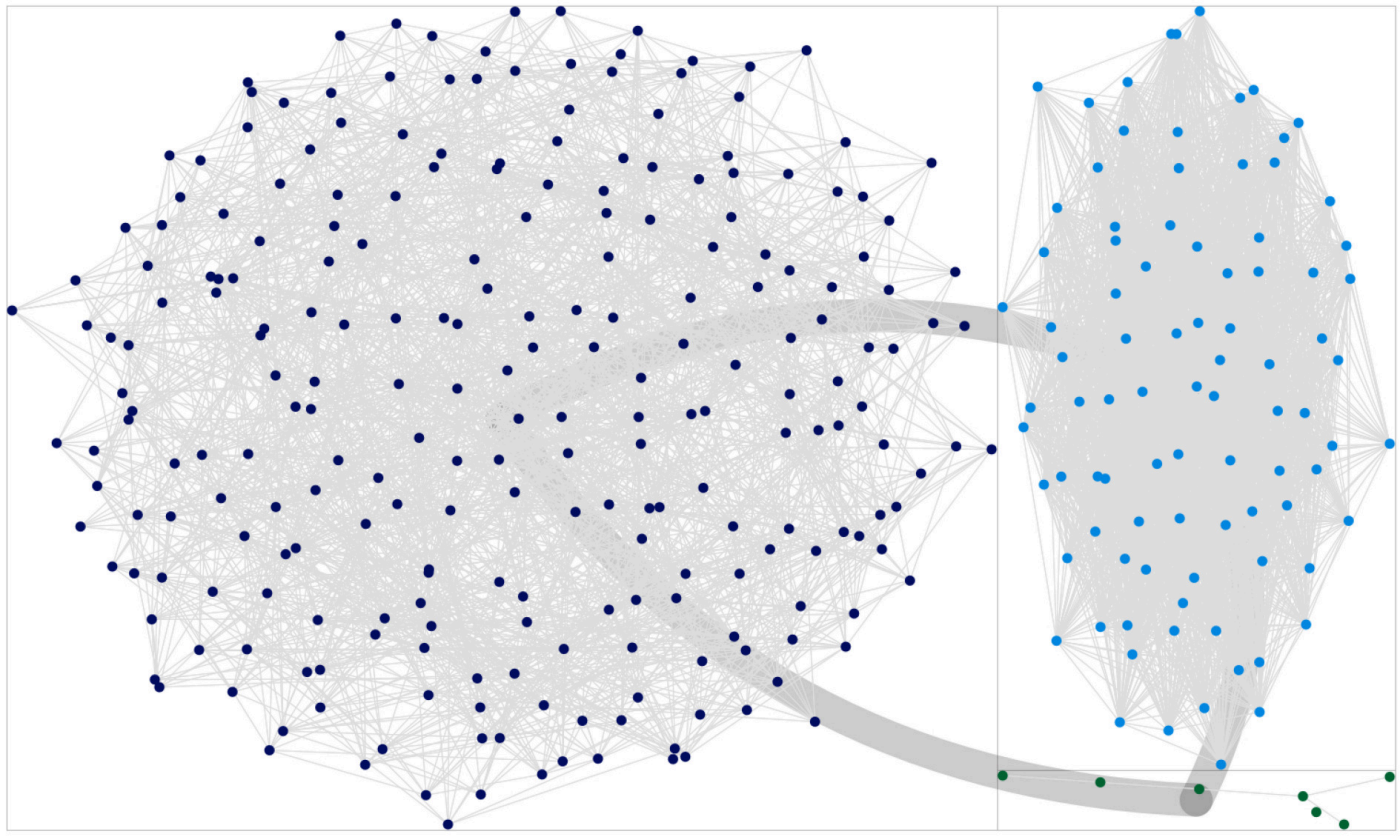
**Plot of the clusters obtained via the CNM algorithm, for the network reconstructed using a semi-observational LBP approach, with parameters ***Q*** = 5 and ***t***_***m***_ = 0.35**. It is evident how the bigger community (dark blue dots) is overestimated compared to standard approaches (see also Figure 4), at the expense of underestimating minor communities. As stated in the text, this effect can be reduced by lowering *t*_*m*_.

Moreover, it must be remembered how this analysis is affected by an important bias. States other than the active state (i.e., *x*_*i*_ = 1 for node *i*) are assigned randomly, therefore favoring communities of homogeneous cardinality: some misclassified nodes originally belonged to mid-size communities, but at their expense, these nodes where assigned to smaller groups. Networks obtained with very low thresholds are particularly prone to this effect. Some other misclassifications are due to a specular effect: similarly to the “*rich gets richer*” phenomenon, discovery of hidden links increases the size of the major communities at the expense of the smallest ones, as it is expected when modularity-based community detection algorithms are applied to very dense networks. Indeed, in all LBP-reconstructed networks the group of senators members of the party leading the Cabinet was always (mistakenly?) bigger than expected. This effect is evident comparing Figure 4 with Figure 5, where the last one has been indeed obtained with LBP and a relatively high *t*_*m*_ = 0.35. The specular consideration above suggests how to compensate this artifact, by lowering opportunely the threshold *t*_*m*_ (the corresponding network graph is omitted for brevity). In any case, it shall be remembered how major Senate groups were actually bigger at the beginning of the legislature, compared to its end (when a few independent groups had been founded). By inferring weak links, it can be thus argued how LBP algorithms thus proved more efficient in merging communities into a few principal components, compared to forcing modularity algorithms to split the network in 2–3 groups (i.e., forcing the CNM algorithm to merge further the 5 communities detected as optimal by its modularity maximization procedure, leading to the results in Figure 3).

On the other side, further increasing the value of *Q* required to rely upon a Generative model, in order to have a number of samples sufficient for the analysis (100 k samples), whereas the reduced number of original data was prone to cause difficulties^27^ in the numerical simulations. As envisaged in Section 2.2, moving from a (Pseudo-)Observational to a Generative approach produced a degradation in the results, because of losing the temporal information of the available data. In conclusion, an approach with higher *Q*, but still low enough to be based upon observational data, seems to produce the best and more stable results for both hidden links and community detection purposes.

## 4. Conclusions

Along the paper, a method based upon Q-state Potts inverse problem and Bethe-LBP approximation for network reconstruction was elucidated. Several possible ways were disclosed, to use the method for inferring links among the nodes of a generic networked social structure, under the hypotheses that: (i) actions like roll call sponsorships resemble decision-making processes and (ii) that these processes can be modeled efficiently by methods used for inferring the structure of an ensemble of quantum states, observed repeatedly over time.

The LBP-based resolution of the inverse problem was applied for the first time to reconstruct a generic graph structure. More specifically, in the Social Sciences realm, this work has been the first to use a Q-state model (instead of Ising model) to infer the structure of a real network. The study case chosen was the Italian Senate, analyzed starting from a dataset tracking law proposal co-sponsorships. This allowed to evaluate the power of the method in detecting those links, that cannot be retrieved via standard reconstruction methods. Also the role of the diverse modeling choices—and peculiar parameters employed—was thoroughly discussed, finding how the maximal value of *Q* permitted by the Potts model can introduce crucial differences in the quality of the results, alongside with aprioristic knowledge about the network structure.

It was investigated, as well, the capability of the model to reproduce the community structure of the network and the single memberships of the senators: it was found that the present method must be carefully reviewed, compared to standard ones, in order to produce a reliable output. In fact, a naive application without any further assumption may lead to completely wrong conclusions. The reason is that the Potts-LBP method is much closer to an ab-initio approach, therefore it originally embeds no information such as the weight to be assigned to inactive vs. active states, or direct vs. indirect correlations, or how weak connections shall be considered *noisy*, …In turn, this higher flexibility allows to explore the role (and therefore the plausibility) of several assumptions made when reconstructing the network.

The authors envisage how interesting directions for further investigation may be the adoption of a full quantum treatment of the Potts model, as well as the possibility to apply this extended method to cases where data retrieved for the network do exhibit natively non-bipartite features, thus allowing a more direct application of generic Q-state Potts models.

## Funding

This work was part of the project “MUSCA” (PAC02L1-0018), funded by the Italian Ministry of Education, University and Research.

### Conflict of interest statement

The authors declare that the research was conducted in the absence of any commercial or financial relationships that could be construed as a potential conflict of interest.

## Footnotes

^1^Adopted elsewhere in SNA literature for the same task.

^2^Publicly available at http://www.senato.it/leg/16/BGT/Schede/Attsen/Sena.html.

^3^See Section 2.

^4^To be intended as those states, that label nodes observed to be inactive, whereas certain other activities are being performed by other nodes.

^5^E.g., the maximum number of allowed states is intrinsically limited to 2, while a generic Q-states Potts model allows *Q* ∈ N, *Q* ≥ 2. Indeed, in Section 3, it will be shown how an inverse Ising problem fails for our case study.

^6^To be specific, the observations are supposed to be sampled from a certain MRF.

^7^I.e., they cannot be observed simultaneously for the same object.

^8^Here and in the following single-node terms are skipped for simplicity.

^9^And in particular, it is unknown which nodes in the interaction model *E* are truly linked to each other, i.e., have a non-negligible interaction: (*i, j*) ∈ *E* ⇔ *H*_(*i, j*)_ ≇ 0.

^10^I.e., assuming that $J_{\left(i,j\right)}^{\left(k,l\right)}\propto \delta \left(k,l\right)$, where δ(*k, l*) is the *Kronecker delta*. Indeed, also in the *pseudo-observational* models defined below, the parameters α_(*i, j*)_ act as an initial guess for the interactions, based upon the assumption that interactions shall be inferred only when simultaneous participation in activities occurs, but this is tested against the observations.

^11^Usually one or just a few. In some special cases, the law undergoes a peculiar path where no initial senator is quoted for sponsoring the proposal.

^12^Corresponding to the first Cabinet.

^13^Indeed, when in the following there will be references to the “true” memberships of the senators, these have been deduced by the participation of the senators to political *groups*: a specificity of the Italian Parliament, that enables the tracking of a senator's loyalty to a party or group of parties. See http://www.senato.it/leg/16/BGT/Schede/GruppiStorici/Grp.html. When, along with the period under observation, a senator belonged to multiple groups, he/she was assigned to the group where he spent most of the observation time.

^14^Actually, a specular interpretation is that the mechanism used to track interaction was poorly efficient, and therefore links in excess shall be excluded by an inference scheme.

^15^As we will call them in the following for simplicity.

^16^In the following for simplicity: *active correlations*.

^17^Here by *small*, we intend parties with a number of senators below the threshold of 10, because this is the minimum number to constitute an official *group* in the Italian Senate. Smaller parties are obliged to group in the so called *mixed group*.

^18^E.g., frequency considerations, as those used in the following Equations (11) and (13) for the semi-observational model.

^19^Used in a second step to calculate the probability of observations in the sample, see Equation (12).

^20^I.e., there is no temporal allocation of the single observations, but only a global count.

^21^Indeed, once the preliminary model has been decided, samples can be drawn from it in abundance, whereas a real sampling of a social network is clearly bound to pragmatic constraints.

^22^For the case *Q* = 2, actually, it is advised to adopt a fictitious α = constant ≪ 1, because the critical value for the 2-state Potts model is α = 0.88, therefore the calculation may turn unstable if averages are computed directly.

^23^Clearly, the density and thus the number of links detected in the Jaccard network is independent from any threshold chosen.

^24^This can be inferred by recalling that the probability to observe a certain collective state *x* has the form in Equation (4).

^25^Here and in the following, communities are always detected with a very successful method based upon random graph theory, the Clauset-Newman-Moore (CNM) method (Clauset et al., 2004). Only communities whose sizes are bigger than 3 nodes will be considered, while isolated nodes and dyads will be omitted.

^26^Preserving more links, indeed, leads to the discovery of weak interactions.

^27^The simulation for high values of *Q* requires limited precision in the intermediate values calculated, to reduce the memory space required.
